# Functional Interpretation of Recurrent Genetic Variants in Hepatocellular Carcinoma: Molecular Consequences and Clinical Relevance

**DOI:** 10.1155/humu/6223429

**Published:** 2026-06-03

**Authors:** Yuntao Ye, Zhulin Xu, Jiang Wang, Chunyu Chen, Yuan Peng, Bo Li

**Affiliations:** ^1^ Department of General Surgery (Hepatopancreatobiliary Surgery), The Affiliated Hospital of Southwest Medical University, Luzhou, China, ahswmu.cn; ^2^ Academician (Expert) Workstation of Sichuan Province, Metabolic Hepatobiliary and Pancreatic Diseases Key Laboratory of Luzhou City, The Affiliated Hospital of Southwest Medical University, Luzhou, China, ahswmu.cn; ^3^ Department of Laboratory Medicine, Southwest Medical University, Luzhou, China, swmu.edu.cn; ^4^ Chengdu University of Traditional Chinese Medicine, Chengdu, China, cdutcm.edu.cn; ^5^ Department of General Surgery, Dazhou Central Hospital, Dazhou, China, dzcch.com; ^6^ Department of Oncology, Chongqing Academy of Medical Sciences, Chongqing General Hospital, Chongqing University, Chongqing, China, cqu.edu.cn

**Keywords:** biomarkers, driver mutations, functional interpretation, hepatocellular carcinoma, multiomics, precision oncology, recurrent genetic variants, tumor microenvironment

## Abstract

Hepatocellular carcinoma (HCC) is characterized by substantial molecular heterogeneity shaped by recurrent genetic alterations. Large‐scale genomic studies have defined the mutational landscape of HCC, but the biological interpretation and clinical utility of these variants remain incompletely established. Increasing evidence suggests that key driver alterations influence tumor behavior not only through direct pathway dysregulation but also through downstream transcriptional programs, epigenetic remodeling, proteomic changes, metabolic adaptation, and tumor–immune interactions. Therefore, variant interpretation in HCC requires a framework that extends beyond mutation frequency and integrates functional annotation, multiomics profiling, experimental validation, and clinical evidence. In this review, we summarize major recurrent genetic alterations in HCC, including variants affecting telomere maintenance, cell cycle control, WNT/*β*‐catenin signaling, and chromatin remodeling. We then discuss how these alterations shape molecular phenotypes across transcriptomic, proteomic, metabolic, and immune layers. Particular attention is given to distinguishing well‐supported variant–phenotype relationships from emerging or exploratory associations. We further evaluate the translational relevance of recurrent variants in diagnosis, prognosis, liquid biopsy, and therapeutic stratification while emphasizing that most variant‐informed applications in HCC remain investigational rather than clinically validated. Finally, we discuss current challenges and future directions, including functional genomics, single‐cell and spatial profiling, and prospective clinical validation. This review proposes a variant‐centered interpretive framework for understanding how recurrent genetic alterations contribute to HCC biology and how they may inform future precision oncology strategies.

## 1. Introduction

Hepatocellular carcinoma (HCC) represents one of the most genetically heterogeneous malignancies and remains a leading cause of cancer‐related mortality worldwide [1, 2]. Advances in next‐generation sequencing over the past decade have substantially expanded our understanding of the genomic landscape of HCC, revealing a spectrum of recurrent somatic alterations affecting genes involved in cell cycle regulation, chromatin remodeling, telomere maintenance, and oncogenic signaling pathways [3, 4]. Large‐scale efforts such as The Cancer Genome Atlas (TCGA) and the International Cancer Genome Consortium (ICGC) have shown that HCC tumors share several recurrent driver events, yet individual tumors often harbor distinct combinations of genetic alterations. This diversity contributes to marked intertumoral variability in biological behavior, molecular subtype, immune contexture, and therapeutic response [5, 6].

Despite these advances in genomic characterization, translating variant information into biological insight and clinical utility remains a major challenge [7, 8]. Many recurrent mutations have been cataloged, but their functional consequences are often incompletely understood, particularly when variant effects are mediated through transcriptional states, epigenetic regulation, metabolic dependencies, or tumor microenvironment interactions [9]. Mutation frequency alone is, therefore, insufficient to determine biological importance or clinical relevance. A low‐frequency alteration may have strong functional consequences in a defined molecular context, whereas a recurrent event may still require additional evidence before it can be interpreted as clinically actionable. As a result, the interpretation of genetic variants has gradually shifted from simple mutation annotation toward understanding their broader molecular and phenotypic consequences.

A variant‐centered framework for HCC should integrate several layers of evidence rather than treat mutations as isolated genomic events. First, recurrent variants need to be annotated according to their predicted functional impact, affected pathway, recurrence across cohorts, and evidence supporting driver status. Second, these variants should be linked to downstream molecular phenotypes, including transcriptional programs, epigenetic states, protein‐level pathway activity, metabolic remodeling, and tumor immune features. Third, the strength of evidence should be considered hierarchically. Associations supported only by computational prediction or retrospective cohort analysis should be distinguished from those supported by mechanistic experiments, independent validation cohorts, or prospective clinical evidence. This distinction is especially important in HCC, where many variant–phenotype relationships are biologically plausible but not yet clinically validated.

Within this framework, different recurrent alterations carry different interpretive weight. TP53 mutations are strongly associated with impaired genome surveillance, chromosomal instability, and aggressive tumor biology. CTNNB1 and other WNT/*β*‐catenin pathway alterations define a biologically recognizable subgroup with characteristic transcriptional and immune features, although their role as predictive biomarkers for immunotherapy remains under investigation. TERT promoter mutations represent early events that support telomerase reactivation and cellular immortalization, whereas alterations in chromatin remodeling genes such as ARID1A and ARID2 suggest links between genetic disruption, chromatin accessibility, and transcriptional plasticity. Thus, the goal of variant interpretation is not only to identify which genes are mutated but also to determine which molecular consequences are robust, which remain exploratory, and which may eventually inform clinical decision‐making.

Recent progress in multiomics profiling has provided new opportunities to implement this interpretive framework. Integrative analyses combining genomic, transcriptomic, epigenomic, proteomic, metabolomic, single‐cell, and spatial data have begun to reveal how recurrent variants influence tumor biology beyond their immediate genomic context. Specific driver alterations have been associated with transcriptional subclasses, immune microenvironment patterns, metabolic states, and pathway activity profiles. Importantly, these molecular layers should not be viewed as parallel observations. Instead, they can be integrated into a genotype‐to‐phenotype model in which genetic variants initiate molecular cascades that are modified by epigenetic regulation, protein‐level signaling, metabolic adaptation, and microenvironmental feedback.

Understanding the molecular consequences of genetic variants is not only a biological question but also a translational necessity [10]. Variant‐associated molecular features have increasingly been explored as potential biomarkers for diagnosis, prognosis, and therapeutic stratification [11]. However, their clinical interpretation requires caution. In current HCC practice, most recurrent somatic variants are not yet established as routine treatment selection biomarkers. For example, certain genomic alterations have been associated with immune phenotypes or differential treatment response in retrospective or exploratory analyses [12, 13], but robust clinical implementation still requires prospective validation, standardized assays, and clear evidence that variant‐informed decisions improve patient outcomes. Therefore, distinguishing clinically actionable biomarkers from candidate markers and conceptual future applications is essential [14].

In this context, a variant‐centered perspective that integrates molecular consequences across multiple biological layers may provide a more effective strategy for interpreting genetic alterations in HCC. Rather than considering mutations solely as static genomic events, emerging evidence supports viewing them as initiators of dynamic molecular processes that influence tumor cell states, microenvironment interactions, and disease trajectories. Such a framework may facilitate the identification of biologically informed biomarkers and improve the translation of genomic findings into clinically relevant applications.

This review summarizes current knowledge on recurrent genetic variants in HCC, with a focus on their molecular consequences and clinical relevance. We first discuss major recurrent alterations affecting telomere maintenance, cell cycle control, WNT/*β*‐catenin signaling, chromatin remodeling, and genome stability. We then examine how these variants shape transcriptomic, proteomic, metabolic, epigenetic, and immune phenotypes through integrative molecular profiling. Finally, we evaluate their translational relevance in diagnosis, prognosis, liquid biopsy, and therapeutic stratification while emphasizing the distinction between established clinical applications and emerging investigational opportunities. By connecting genetic alterations with molecular phenotypes and clinical evidence, this review is aimed at providing a structured framework for functional variant interpretation in HCC.

## 2. Recurrent Genetic Alterations in HCC

Comprehensive genomic profiling studies have revealed that HCC is characterized by recurrent alterations affecting a limited number of biologically dominant processes rather than a large number of highly recurrent individual genes [15, 16]. These alterations primarily converge on pathways regulating genome stability, developmental signaling, chromatin organization, and cellular immortality [17]. From a functional interpretation perspective, these mutation classes should be prioritized according to their recurrence, mechanistic support, downstream molecular consequences, and clinical relevance. Among them, TERT promoter mutations, TP53 mutations, and CTNNB1/WNT/*β*‐catenin pathway alterations represent the most consistently observed and best‐characterized events in HCC, whereas alterations in chromatin remodeling genes and other genome maintenance regulators are less frequent but may exert important context‐dependent effects. This prioritization provides a more useful framework than simply listing mutation frequencies, because different variant classes differ substantially in biological weight, interpretive confidence, and clinical maturity [18].

### 2.1. Variants Affecting Cell Cycle Regulation and Genomic Stability

Disruption of cell cycle control and genome maintenance mechanisms represents a central feature of HCC tumorigenesis [19]. Among these alterations, TP53 mutations are the most extensively studied and are detected in approximately 25%–35% of HCC cases, although the reported frequency varies by etiology, geographic background, sequencing strategy, and cohort composition [20]. TP53 encodes a critical tumor suppressor involved in DNA damage response, apoptosis regulation, and cell cycle arrest [21]. Loss of TP53 function is frequently associated with increased chromosomal instability and more aggressive tumor phenotypes [22, 23]. Compared with many lower frequency alterations, the biological consequences of TP53 disruption are relatively well established; however, its role should be interpreted primarily as a marker of aggressive tumor biology and genomic instability rather than as a validated treatment selection biomarker in routine HCC practice.

Other alterations affecting genome maintenance pathways have also been described, although they occur at lower frequencies and are often more difficult to interpret individually. Rather than acting as isolated determinants, these alterations may cooperate with TP53 loss, copy number changes, replication stress, or mutational processes to increase genomic diversity and tumor evolution. Tumors harboring defects in genome stability pathways often exhibit higher proliferative capacity and altered stress‐response programs [24]. However, the functional relevance of many non‐TP53 genome maintenance alterations remains context‐dependent and usually requires integration with allelic status, copy number burden, transcriptional programs, and experimental validation. Thus, genome instability in HCC should be interpreted as a composite phenotype shaped by both recurrent driver mutations and broader genomic architecture.

### 2.2. Alterations in WNT/*β*‐Catenin Signaling

Aberrant activation of the WNT/*β*‐catenin signaling pathway represents another major molecular feature of HCC [25]. Activating mutations in CTNNB1, which encodes *β*‐catenin, as well as inactivating alterations in negative regulators such as AXIN1, contribute to pathway dysregulation in a significant fraction of tumors [26]. Among recurrent HCC alterations, CTNNB1 mutations have one of the clearest genotype–phenotype relationships, because they directly stabilize *β*‐catenin and activate WNT‐dependent transcriptional programs. These tumors are frequently associated with hepatocyte differentiation–related signatures, distinct transcriptional subclasses, and biologically recognizable tumor subgroups [27, 28].

The biological relevance of WNT/*β*‐catenin activation extends beyond tumor cell–intrinsic transcriptional regulation. Tumors with activated WNT/*β*‐catenin signaling often show distinctive tumor microenvironment features [13, 29, 30]. Several studies have suggested that this molecular subtype may be associated with reduced immune cell infiltration and altered inflammatory signaling, indicating that oncogenic pathway activation may indirectly shape immune phenotypes [31]. Nevertheless, this relationship should not be overgeneralized. WNT/*β*‐catenin activation may contribute to immune exclusion in certain molecular contexts, but immune responsiveness is also shaped by tumor etiology, antigen presentation, co‐occurring alterations, stromal composition, and treatment setting. These findings highlight how driver alterations may influence tumor biology not only through intrinsic tumor cell effects but also through interactions with the surrounding microenvironment.

### 2.3. Chromatin Remodeling Gene Alterations

Epigenetic dysregulation is increasingly recognized as an important contributor to HCC development [32]. Mutations affecting chromatin remodeling genes, particularly ARID1A and ARID2, have been identified in a subset of tumors. These genes encode components of the SWI/SNF ATP‐dependent chromatin remodeling complex, which regulates nucleosome positioning, chromatin accessibility, enhancer activity, and transcriptional control. Disruption of SWI/SNF components may, therefore, reshape the regulatory landscape of tumor cells and lead to broad transcriptional reprogramming rather than activation of a single linear signaling pathway [33, 34].

The functional consequences of chromatin remodeling alterations are likely to be highly context‐dependent. By altering chromatin accessibility and transcriptional permissiveness, ARID1A or ARID2 disruption may facilitate tumor plasticity, adaptive cell state transitions, and responses to environmental stress, including hypoxia, inflammation, and therapeutic pressure. Compared with TP53, CTNNB1, and TERT promoter alterations, however, the clinical interpretation of ARID1A/ARID2 mutations remains less mature. Their biological relevance is increasingly supported, but their prognostic or predictive value has not been consistently established across HCC cohorts. For this reason, chromatin remodeling alterations should be interpreted through integration with epigenomic profiles, transcriptomic states, immune or stromal phenotypes, and functional validation.

### 2.4. Variants Involved in Telomere Maintenance and Cellular Immortalization

Telomere maintenance represents another key process frequently altered in HCC [35]. Mutations in the promoter region of TERT, which encodes the catalytic subunit of telomerase, are among the most common genetic events identified in liver cancer. The two major hotspot mutations, C228T and C250T, occur upstream of the TERT transcription start site and generate de novo binding motifs for ETS family transcription factors. These newly created regulatory motifs enhance TERT promoter activity, increase TERT transcription, and promote telomerase reactivation. Through this mechanism, TERT promoter mutations support telomere maintenance, replicative immortality, and clonal expansion during hepatocarcinogenesis [24, 36].

TERT promoter mutations are particularly notable because they may occur early during hepatocarcinogenesis and have also been detected in premalignant liver lesions [37]. This timing suggests that telomerase reactivation may function as an early enabling event rather than merely a late progression feature. Mechanistically, TERT promoter mutations are, therefore, important not only because they are frequent but because they connect a noncoding regulatory alteration to transcriptional activation, cellular immortalization, and early tumor evolution. Despite their high prevalence, the clinical implications of TERT promoter mutations remain incompletely defined [38]. At present, they are best interpreted as early and biologically important alterations with potential relevance to early detection, risk stratification, or liquid biopsy–based monitoring, rather than as established predictors of systemic therapy response.

### 2.5. From Mutation Catalogs to Functional Interpretation

Although the major recurrent alterations in HCC have now been extensively described, a central challenge remains in understanding how these variants translate into tumor phenotypes [39]. The presence of a mutation alone does not fully explain tumor behavior, as the biological impact of genetic alterations often depends on downstream molecular consequences and interactions with other genomic events [40, 41]. Accordingly, variant interpretation should distinguish robust genotype–phenotype relationships from exploratory associations. TP53‐associated genomic instability, CTNNB1‐associated WNT pathway activation, and TERT promoter mutation–associated telomerase reactivation represent relatively well‐supported examples. In contrast, many links between lower frequency variants, metabolic states, immune phenotypes, or therapeutic response remain hypothesis‐generating and require further validation.

**Figure 1:**
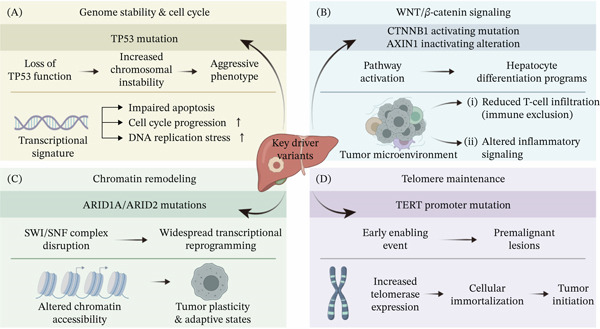
Major recurrent genetic variants in HCC and their multilayer molecular consequences. In each panel, the top section denotes the primary genomic alteration, while the bottom highlights downstream molecular and microenvironmental effects. (A) TP53 mutations induce chromosomal instability and aggressive phenotypes, driving distinct transcriptional signatures including impaired apoptosis. (B) CTNNB1 or AXIN1 alterations activate WNT signaling to alter hepatocyte differentiation, ultimately promoting an immune‐excluded tumor microenvironment. (C) ARID1A/ARID2 mutations disrupt the SWI/SNF complex and alter chromatin accessibility, enabling widespread transcriptional reprogramming and adaptive tumor plasticity. (D) TERT promoter mutations act as early enabling events in premalignant lesions, increasing telomerase expression to drive cellular immortalization and tumor initiation.

This functional perspective emphasizes the need to move beyond mutation catalogs toward integrated biological interpretation. Recurrent variants may act as upstream determinants of transcriptional programs, metabolic states, chromatin organization, and tumor microenvironment interactions, but these effects are rarely captured by mutation status alone. Integrative molecular analyses can help reconstruct the path from genotype to phenotype by linking variant classes to downstream molecular consequences and by clarifying which associations are mechanistically supported, clinically relevant, or still exploratory. These considerations provide the rationale for examining the molecular consequences of HCC variants across multiple biological layers. In the following section, we discuss how integrative molecular studies have begun to clarify these downstream effects and how they may inform functional interpretation of recurrent genetic alterations in HCC (Figure 1).

## 3. Molecular Consequences of Recurrent Genetic Variants in HCC

While recurrent genetic alterations provide a framework for understanding HCC tumorigenesis, their biological significance ultimately depends on their downstream molecular consequences [15, 42–44]. A recurrent variant does not necessarily produce a single fixed phenotype. Instead, its functional impact is shaped by transcriptional regulation, chromatin context, protein‐level signaling, metabolic adaptation, and tumor microenvironment feedback. Increasing evidence suggests that driver alterations do not act in isolation but reshape tumor biology through coordinated effects on gene expression programs, protein networks, metabolic states, and immune or stromal interactions [45, 46]. Understanding these downstream effects is, therefore, essential for interpreting how genetic variants contribute to tumor heterogeneity and clinical behavior [39, 47].

### 3.1. Transcriptomic Consequences of HCC Driver Variants

One of the most direct consequences of recurrent genetic alterations is the reprogramming of transcriptional landscapes [27, 48, 49]. Integrative analyses combining genomic and transcriptomic data have demonstrated that key driver mutations in HCC are frequently associated with distinct gene expression patterns that reflect underlying pathway activation and cellular state transitions [50]. However, transcriptomic changes should not be interpreted as simple downstream readouts of mutation status alone. They are also modified by DNA methylation, chromatin accessibility, enhancer usage, histone modification, and the broader epigenetic state of the tumor cell. Therefore, integrating genomic and epigenomic information is important for understanding why tumors with similar driver alterations may still display different biological phenotypes.

For example, TP53‐mutant tumors often exhibit transcriptional signatures associated with cell cycle progression, DNA replication stress, and impaired apoptotic regulation [51]. These transcriptional programs are consistent with the canonical role of TP53 in maintaining genomic integrity and suggest that loss of TP53 function may promote proliferative advantages through coordinated transcriptional deregulation [52]. In this setting, the transcriptional consequences of TP53 loss are relatively well supported, particularly with respect to proliferative and genome‐instability phenotypes. Nevertheless, TP53 mutation alone does not fully explain the diversity of downstream tumor states, because co‐occurring copy number alterations, chromosomal instability, and epigenetic remodeling may further modify gene expression output. Similarly, alterations in chromatin remodeling genes such as ARID1A may influence global transcriptional architecture by modifying chromatin accessibility, thereby enabling tumor cells to adopt adaptive transcriptional states [53, 54].

In contrast, CTNNB1‐mutant tumors frequently display transcriptional profiles indicative of WNT pathway activation and hepatocyte differentiation programs [55]. These tumors often exhibit distinct molecular characteristics compared with TP53‐driven tumors, reinforcing the concept that different driver alterations may define biologically distinct transcriptional subclasses of HCC. This represents one of the clearer genotype–transcriptome relationships in HCC, because CTNNB1 alterations directly converge on *β*‐catenin stabilization and WNT‐dependent transcription. Even so, the downstream phenotype may vary depending on AXIN1 status, liver disease etiology, tumor differentiation state, and interaction with other molecular layers. Such observations suggest that transcriptional consequences of genetic variants may provide a more functional readout of tumor biology than mutation status alone.

Recent advances in single‐cell transcriptomics have further refined this understanding by revealing how genetic alterations may influence cellular composition and cell state heterogeneity [56, 57]. Bulk transcriptomic analysis may obscure whether a variant‐associated signature reflects intrinsic changes in tumor cells, expansion of a specific malignant subclone, infiltration by stromal or immune cells, or a mixture of these processes. Single‐cell analyses have demonstrated that transcriptional programs linked to tumor progression, proliferation, immune evasion, or metastatic potential may be unevenly distributed across tumor cell populations [58]. This helps explain why the same driver alteration may be associated with different phenotypic outputs across patients or even within different regions of the same tumor. These findings highlight the importance of considering cellular heterogeneity when interpreting the functional consequences of HCC variants.

### 3.2. Proteomic and Metabolic Consequences of Genetic Alterations

Beyond transcriptional regulation, genetic variants may also influence tumor biology through effects on protein expression networks and metabolic pathways. Proteomic analyses have demonstrated that genomic alterations do not always translate directly into corresponding transcript‐level changes, emphasizing the importance of examining protein‐level consequences to fully understand variant function [59]. This distinction is particularly important in signaling‐driven tumors, where phosphorylation, ubiquitination, acetylation, protein stabilization, and altered degradation can determine pathway activity more directly than mRNA abundance.

For instance, alterations affecting WNT signaling components may lead to downstream changes in proteins involved in cell adhesion, cytoskeletal organization, and signal transduction [60]. Similarly, TP53‐deficient tumors have been associated with altered expression of proteins involved in stress response and metabolic regulation [61]. These examples illustrate that proteomic profiling can refine genotype–phenotype interpretation by identifying activated pathways, compensatory signaling networks, or protein‐level vulnerabilities that are not apparent from transcriptomic data alone. Phosphoproteomic analysis may be especially informative for evaluating kinase signaling, stress‐response pathways, and therapeutic dependencies associated with specific variant contexts. Thus, protein‐level profiling provides an essential intermediate layer between genetic alteration and cellular phenotype.

Metabolic reprogramming also represents an important downstream consequence of genetic alterations in HCC [62]. Several studies have suggested that specific driver mutations may be associated with distinct metabolic phenotypes, including alterations in lipid metabolism, oxidative phosphorylation, and glycolytic activity. These metabolic states are unlikely to be determined by mutation status alone; rather, they emerge from interactions among oncogenic signaling, liver‐specific metabolic programs, nutrient availability, hypoxia, mitochondrial function, and microenvironmental constraints. Such metabolic differences may reflect adaptive responses to oncogenic signaling and microenvironmental constraints. Importantly, these metabolic features may also create potential therapeutic vulnerabilities, highlighting the translational relevance of understanding variant‐associated metabolic states [63]. However, variant‐associated metabolic phenotypes should be interpreted cautiously, because many reported associations are derived from retrospective omics analyses and remain insufficiently validated in prospective or functional studies.

### 3.3. Influence of Genetic Variants on the Tumor Immune Microenvironment

An emerging area of interest is how genetic alterations shape interactions between tumor cells and the immune microenvironment [64, 65]. Increasing evidence suggests that driver mutations may indirectly influence immune cell infiltration, inflammatory signaling, and immune evasion mechanisms [13, 66].

For example, activation of the WNT/*β*‐catenin pathway has been associated with reduced T‐cell infiltration and features consistent with immune exclusion in several tumor types, including HCC [31, 67]. This observation suggests that oncogenic pathway activation may influence tumor immune accessibility through effects on chemokine signaling or stromal interactions [29, 68]. Nevertheless, WNT/*β*‐catenin activation should not be considered a universal marker of immunotherapy resistance. The immune phenotype of a given tumor may also depend on viral etiology, tumor mutational processes, antigen presentation capacity, vascular and stromal organization, myeloid cell composition, and prior or concurrent treatment. Conversely, tumors with TP53 alterations may exhibit different immune characteristics, potentially reflecting the impact of genomic instability on immune recognition processes [69].

Single‐cell and spatial approaches are particularly useful for refining these immune‐related interpretations. They can identify which cell populations produce chemokines or immunosuppressive ligands, which immune subsets are excluded or dysfunctional, and how malignant cells communicate with macrophages, cancer‐associated fibroblasts, endothelial cells, and T cells. Ligand–receptor and cell–cell communication analyses may reveal, for example, whether a variant‐associated malignant subpopulation is linked to fibroblast activation, myeloid recruitment, T‐cell exhaustion, or altered antigen presentation niches. These methods, therefore, help move variant interpretation from bulk immune scores toward ecosystem‐level mechanisms.

Taken together, these observations support the concept that genetic variants may contribute to shaping tumor immune phenotypes through complex regulatory networks rather than direct immune gene mutations. At present, the immune implications of recurrent variants are best viewed as biologically informative and potentially useful for stratification but not yet sufficient as standalone clinical biomarkers for immunotherapy selection. This restrained interpretation is particularly important when translating molecular associations into therapeutic implications.

### 3.4. Integrative Perspectives on Variant‐Driven Molecular Phenotypes

Taken together, these findings highlight that recurrent genetic variants may exert broad effects across multiple molecular layers. Rather than acting solely as genomic events, driver alterations may function as upstream regulators that shape tumor phenotypes through coordinated effects on gene expression, protein networks, metabolism, and tumor microenvironment interactions. A unified model of variant interpretation should, therefore, connect genotype to phenotype through a sequence of interacting layers: genetic alteration, regulatory and epigenetic context, transcriptional output, protein‐level pathway activity, metabolic adaptation, cellular state diversity, and immune or stromal remodeling.

This integrative perspective suggests that understanding the biological significance of genetic variants requires more than identifying mutation status. For well‐established relationships, such as TP53‐associated genomic instability, CTNNB1‐associated WNT activation, and TERT promoter mutation–associated telomerase reactivation, multiomics data can refine mechanistic interpretation and identify downstream phenotypes. For emerging relationships, particularly those involving metabolism, immune contexture, and therapeutic response, integrative profiling should be used to generate and prioritize hypotheses that require further functional and clinical validation. By linking genetic alterations to molecular phenotypes, integrative analyses may provide a more comprehensive framework for interpreting variant function and identifying biologically meaningful tumor subtypes.

## 4. Functional Interpretation of HCC Variants Through Integrative Molecular Profiling

**Figure 2:**
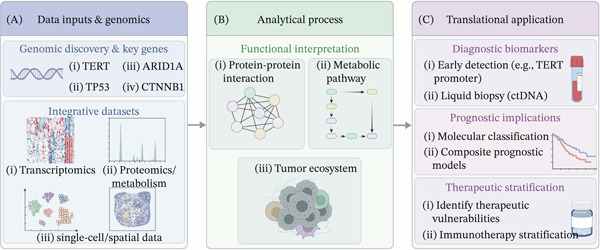
Integrative framework for the functional interpretation and translational application of HCC variants. This workflow illustrates the transition from genomic discovery to clinical utility through multiomics integration. (A) Data inputs: Key recurrent alterations (e.g., TERT and TP53) are integrated with transcriptomic, proteomic/metabolic, and single‐cell/spatial datasets. (B) Analytical process: Moving beyond simple annotation, integrative analyses construct mechanistic models—including molecular networks and tumor ecosystems—to interpret functional consequences. (C) Translational application: Derived molecular features guide clinical decision‐making across diagnostic biomarkers (e.g., ctDNA), prognostic classification, and therapeutic stratification.

Although genomic sequencing has identified a spectrum of recurrent alterations in HCC, understanding their biological significance requires approaches that extend beyond mutation detection alone [70, 71]. A major challenge in cancer genomics is that many recurrent variants lack clear functional annotation, particularly when their effects are mediated through complex regulatory networks [72]. Functional interpretation should, therefore, be viewed as a stepwise process that connects variant detection, variant annotation, molecular phenotype mapping, experimental validation, and clinical evidence assessment. Integrative molecular profiling has, therefore, emerged as an important strategy to connect genetic alterations with downstream biological processes and improve functional interpretation [73, 74] (Figure 2).

### 4.1. Moving Beyond Mutation Catalogs Toward Functional Interpretation

Traditional genomic analyses have largely focused on identifying recurrently mutated genes and estimating their mutation frequencies [75]. While this information provides valuable insights into tumor evolution, it offers limited information regarding how variants influence tumor behavior [76]. A more informative approach begins with systematic variant annotation. Coding variants can be classified according to predicted consequence, affected transcript, protein domain, evolutionary conservation, and known cancer relevance, whereas noncoding variants require assessment of regulatory context, promoter or enhancer location, transcription factor binding, chromatin accessibility, and potential effects on gene expression. Commonly used annotation platforms, such as ANNOVAR, Ensembl Variant Effect Predictor, and SnpEff [77–79], can support the initial classification of coding and noncoding variants by mapping them to genes, transcripts, regulatory regions, and predicted functional consequences.

However, annotation alone is not equivalent to biological interpretation. A variant predicted to alter protein sequence or regulatory function still requires additional evidence to determine whether it is a driver event, a passenger alteration, or a context‐dependent modifier of tumor phenotype. Cancer‐specific resources and knowledge bases, including COSMIC, cBioPortal, CIViC, and the Cancer Genome Interpreter, can help place HCC variants into a broader oncologic context by providing information on recurrence, driver evidence, clinical associations, and therapeutic relevance [80–83]. In HCC, this distinction is particularly important because several highly recurrent variants, such as TERT promoter mutations, TP53 mutations, and CTNNB1 mutations, have strong biological support, whereas many lower frequency variants remain incompletely characterized.

An evidence hierarchy is, therefore, needed for variant interpretation. At the lowest level, a variant may be supported only by computational prediction or recurrence in sequencing cohorts. Stronger evidence is provided when the variant is linked to reproducible molecular phenotypes, such as pathway activation, transcriptional subtype, protein‐level signaling, immune contexture, or metabolic state. Even stronger evidence comes from experimental validation using perturbation models, reporter assays, organoids, or patient‐derived systems. The highest level of evidence requires clinical validation, ideally through independent cohorts or prospective biomarker‐stratified studies. This hierarchy helps avoid treating all recurrent variants as equally meaningful and allows robust biological effects to be separated from exploratory associations.

### 4.2. Integrative Analysis Across Molecular Layers

Multilayer molecular profiling approaches have provided important insights into how genetic variants shape tumor biology. Integrative analyses combining genomic and transcriptomic data have demonstrated that many driver mutations are associated with specific expression signatures that reflect pathway activation or suppression [84]. For example, mutation‐stratified differential expression, pathway activity scoring, gene set enrichment analysis, and variant–expression association analysis can be used to determine whether a recurrent alteration is linked to coherent downstream transcriptional programs rather than isolated gene‐level changes. These transcriptional signatures may provide functional evidence supporting the biological impact of genetic alterations [85].

Similarly, integration of genomic and proteomic data has revealed that the downstream effects of mutations are often more accurately reflected at the protein level than at the transcript level [86, 87]. This observation is particularly relevant for signaling pathways in which posttranscriptional regulation plays a significant role. By combining multiple molecular layers, integrative approaches may, therefore, provide a more comprehensive view of variant‐associated biological effects.

The emergence of single‐cell and spatial transcriptomic technologies has further enhanced the ability to interpret genetic variants in the context of tumor ecosystems [88, 89]. These approaches allow investigation of how variant‐associated molecular programs may influence cellular composition, differentiation states, and spatial organization within tumors. Such analyses suggest that the functional consequences of genetic alterations may extend beyond tumor cells themselves and involve broader tumor ecosystem remodeling.

Metabolomic integration adds another layer of functional interpretation, especially in HCC, where liver‐specific metabolic programs strongly influence tumor behavior. Variant‐associated metabolic phenotypes may involve lipid metabolism, bile acid metabolism, oxidative phosphorylation, glycolysis, redox balance, or mitochondrial function. However, these associations should be interpreted cautiously because metabolic states are shaped not only by genetic alterations but also by cirrhosis, viral etiology, hypoxia, nutrient availability, and the surrounding microenvironment. Therefore, metabolomic data are most informative when analyzed together with pathway activity, transcriptomic state, and clinical context rather than as isolated metabolic signatures.

### 4.3. From Molecular Associations to Biological Models

While integrative analyses have identified numerous associations between genetic variants and molecular features, an important next step is the development of biologically meaningful models that explain how these alterations drive tumor phenotypes. Rather than focusing solely on statistical associations, recent studies have emphasized constructing mechanistic frameworks linking genetic alterations to pathway dysregulation and cellular behavior [90].

Such models often conceptualize driver mutations as upstream events that initiate cascades of molecular changes affecting multiple biological processes. For example, a single driver alteration may simultaneously influence transcriptional regulation, metabolic activity, and immune interactions through interconnected signaling networks. This systems‐level perspective may provide a more realistic representation of tumor biology than models based on single pathways [91].

Importantly, these integrative models may also facilitate the identification of variant‐associated vulnerabilities that could be explored for therapeutic purposes. By connecting genetic alterations with functional phenotypes, integrative approaches may help distinguish biologically relevant variants from passenger alterations and prioritize candidates for further investigation.

Together, these observations illustrate that integrative molecular profiling provides a critical bridge between genomic discovery and biological understanding. By enabling functional interpretation of genetic variants, these approaches may improve the identification of biologically meaningful tumor subtypes and support the development of clinically relevant biomarkers. In the following section, we discuss how insights derived from variant interpretation are beginning to inform clinical applications in HCC.

## 5. Translational Relevance of Genetic Variants in HCC

Beyond their biological significance, recurrent genetic alterations in HCC are increasingly being explored for their potential clinical relevance [92, 93]. Understanding how genetic variants influence tumor behavior may provide opportunities for improving diagnosis, risk stratification, and therapeutic decision‐making [94]. However, the translational maturity of genetic variants in HCC is uneven. Some variants have strong biological relevance, some are candidate biomarkers under active investigation, and only a limited number of genomic or molecular features have reached the level of routine clinical actionability. Although the clinical implementation of genomic findings in HCC remains limited compared with some other cancer types, emerging evidence suggests that variant‐informed molecular characterization may contribute to more precise clinical management strategies [95].

### 5.1. Genetic Variants as Diagnostic Biomarkers

Genetic alterations may serve as potential biomarkers for early detection and molecular classification of HCC [96, 97]. Among these, TERT promoter mutations represent one of the most frequently reported genomic events and have been detected not only in established tumors but also in early lesions [98, 99]. This observation suggests that such alterations may represent early molecular events in hepatocarcinogenesis and could potentially be incorporated into molecular diagnostic frameworks [40].

In addition to tissue‐based analyses, advances in liquid biopsy technologies have enabled the detection of tumor‐derived genetic alterations in circulating tumor DNA (ctDNA) [100, 101]. The identification of recurrent variants through minimally invasive approaches has generated interest in their potential application for early detection and disease monitoring [102, 103]. Compared with tissue sequencing, ctDNA has the advantage of repeated sampling and may better capture spatial or temporal tumor heterogeneity. However, ctDNA‐based variant detection in HCC remains technically challenging because tumor fraction can be low, background liver disease may introduce confounding signals, and assay performance may differ across disease stages. While technical and biological challenges remain, these approaches highlight the potential role of genetic variants as accessible molecular markers for clinical evaluation [104].

### 5.2. Prognostic Implications of Recurrent Variants

Several studies have suggested that specific genetic alterations may be associated with clinical outcomes in HCC [105]. For instance, TP53 mutations have frequently been linked to more aggressive tumor behavior and poorer survival, whereas tumors characterized by WNT/*β*‐catenin pathway activation may exhibit distinct clinical trajectories [106, 107]. These observations suggest that genetic alterations may contribute to molecular subclassification of HCC with potential prognostic relevance [108].

However, the prognostic value of individual mutations is often influenced by additional molecular and clinical factors [109]. Increasing evidence indicates that integrating variant information with transcriptomic or clinical features may improve prognostic models compared with mutation status alone [110, 111]. This observation further supports the concept that the clinical relevance of genetic variants is best understood within a broader molecular context rather than as isolated genomic events.

### 5.3. Therapeutic Implications and Precision Oncology Perspectives

The relationship between genetic variants and therapeutic response represents an area of growing interest [112]. Although targeted therapies for HCC remain limited, accumulating evidence suggests that certain molecular alterations may influence treatment sensitivity or resistance [113]. At present, however, most approved systemic therapies for advanced HCC are not selected according to recurrent somatic variants. Major Phase III trials have established the efficacy of multikinase inhibitors and immune checkpoint inhibitor–based regimens, but they generally did not use TP53, CTNNB1, TERT promoter, or ARID1A/ARID2 mutation status as prospective enrollment or treatment selection criteria.

In addition, the association between specific molecular subtypes and immune phenotypes has generated interest in the potential role of variant‐informed stratification in immunotherapy [114, 115]. Tumors characterized by distinct oncogenic signaling patterns may exhibit differences in immune accessibility or inflammatory signaling, which could influence therapeutic responsiveness [116]. Although these associations remain under investigation, they highlight the possibility that variant‐associated molecular features may eventually contribute to treatment stratification strategies.

Despite these advances, translating genomic findings into clinical practice remains challenging. Many variants lack validated therapeutic implications, and functional evidence supporting clinical decision‐making is often limited. These challenges underscore the need for further studies integrating genomic data with functional and clinical evidence.

### 5.4. Distinguishing Clinically Actionable Biomarkers From Candidate Markers

A structured distinction between current clinical actionability and future potential is essential for interpreting HCC variants. Clinically actionable biomarkers should meet several criteria: analytical validity, reproducible association with treatment response or outcome, independent validation, and evidence that biomarker‐guided decisions improve patient management. By this standard, most recurrent somatic variants in HCC are not yet established as routine treatment selection biomarkers.

Candidate biomarkers under investigation include CTNNB1/WNT/*β*‐catenin activation for immune‐excluded phenotypes and possible immunotherapy resistance, TP53 mutation for aggressive biology and prognosis, TERT promoter mutation for early detection or ctDNA‐based monitoring, and chromatin remodeling alterations for tumor plasticity or immune modulation. These markers are biologically informative, but their clinical use remains limited by retrospective evidence, heterogeneous assays, inconsistent cutoffs, and insufficient prospective validation.

Conceptual future applications include variant‐informed multiomics stratification, composite molecular risk models, integration of ctDNA with imaging and serum biomarkers, and functional precision oncology platforms using organoids or patient‐derived models. These approaches may eventually help identify subgroups with distinct prognosis or therapeutic vulnerabilities, but they should currently be framed as research directions rather than established clinical tools.

### 5.5. Current Challenges in Clinical Translation

Several barriers continue to limit the clinical implementation of variant‐based biomarkers in HCC. First, tumor heterogeneity complicates the interpretation of genomic findings, as individual tumors often contain multiple co‐occurring alterations that may influence biological behavior. Second, functional validation of many recurrent variants remains incomplete, limiting confidence in their clinical significance. Third, available clinical trial data rarely include prospective genotype‐defined stratification, making it difficult to determine whether a variant is truly predictive of treatment response or simply associated with broader tumor biology. Fourth, background liver disease, viral etiology, cirrhosis, inflammation, and liver function may confound the relationship between tumor genotype and clinical outcome.

Additionally, the clinical utility of many genomic alterations may depend on their integration with other molecular and clinical variables. This complexity suggests that future clinical applications may require composite biomarker models rather than reliance on single‐gene alterations. For example, mutation status may need to be combined with transcriptomic pathway activity, immune infiltration, ctDNA dynamics, radiological features, pathological variables, and liver function measures to generate clinically useful models. Developing such integrative models will require coordinated efforts combining genomic profiling, functional studies, standardized assays, and prospective clinical validation.

Together, these observations suggest that while the clinical application of variant interpretation in HCC is still evolving, integrative approaches linking genetic alterations with molecular phenotypes may provide a promising path toward more biologically informed clinical decision‐making. The strongest current value of recurrent variants may lie in biological classification, risk modeling, and hypothesis generation, whereas routine treatment selection remains insufficiently supported for most individual variants. Continued efforts to connect genomic discoveries with functional evidence and clinical outcomes will be essential to fully realize the translational potential of variant research in HCC.

## 6. Challenges and Future Perspectives

Despite substantial progress in defining the genomic landscape of HCC, several challenges remain in translating variant discoveries into biological understanding and clinical application [117, 118]. One of the most significant challenges lies in distinguishing biologically meaningful driver alterations from passenger mutations [119, 120]. This distinction cannot be made solely on the basis of mutation frequency. Some recurrent variants may reflect background mutational processes without clear functional consequences, whereas less frequent alterations may have strong biological effects in specific molecular contexts. Although large sequencing efforts have identified numerous recurrent variants, the functional relevance of many alterations remains uncertain. Therefore, future variant interpretation in HCC should combine recurrence, predicted functional impact, pathway relevance, molecular phenotype, experimental evidence, and clinical association rather than relying on genomic annotation alone. This gap highlights the need for systematic functional studies to complement genomic discovery efforts [121, 122].

Tumor heterogeneity represents another major obstacle to variant interpretation [123, 124]. Both intertumoral and intratumoral heterogeneity may influence how genetic alterations manifest at the molecular and phenotypic levels [125]. Even tumors sharing similar driver mutations may display divergent biological behaviors due to differences in cellular composition, epigenetic regulation, or microenvironmental context [126]. This complexity explains why mutation status alone often fails to capture tumor behavior or therapeutic response. A given variant may produce different transcriptional, metabolic, or immune phenotypes depending on the surrounding genomic and cellular ecosystem. These considerations suggest that understanding variant function requires moving beyond tumor cell–intrinsic interpretation and incorporating malignant cell states, stromal components, immune populations, and spatial organization [127].

A further challenge involves the integration of multilayer molecular data into clinically meaningful frameworks [128]. While integrative profiling has revealed important associations between genetic variants and molecular phenotypes, translating these observations into robust biomarkers remains difficult [129]. Many variant‐associated molecular features require validation across independent cohorts and clinical settings before they can be reliably applied in practice [130]. In addition, standardized strategies for integrating genomic and molecular information into clinical workflows remain under development [131].

Another important consideration is the limited availability of functional validation for many recurrent alterations. Computational analyses can generate hypotheses regarding variant function, but experimental validation remains essential for establishing biological relevance. The appropriate validation strategy should depend on the expected mechanism of the variant. For example, promoter mutations may require reporter assays and transcription factor binding studies, signaling alterations may require pathway activity and phosphoproteomic assessment, tumor suppressor alterations may require perturbation models evaluating DNA damage response or cell cycle control, and chromatin remodeling alterations may require assays of chromatin accessibility and transcriptional plasticity. Future studies combining genomic analysis with experimental models will, therefore, be critical for clarifying the functional impact of candidate variants and identifying biologically actionable targets.

Looking forward, several directions may help advance the interpretation of genetic variants in HCC. Increasing integration of functional genomics approaches, including CRISPR‐based screening and perturbation transcriptomics, may help systematically define the biological roles of recurrent alterations [132]. In parallel, continued development of single‐cell and spatial profiling technologies may further clarify how genetic variants shape tumor ecosystems and cellular interactions.

Importantly, future progress will likely depend on closer integration between genomic research and clinical investigation. Establishing clinically annotated molecular datasets and incorporating genomic analysis into prospective studies may help bridge the gap between molecular discovery and clinical application. Such efforts may ultimately enable more biologically informed classification systems and support the development of variant‐informed management strategies.

In summary, significant advances have been made in characterizing the genetic landscape of HCC, but the central challenge has shifted from detecting recurrent variants to interpreting their functional and clinical meaning. A mature framework for HCC variant interpretation should integrate genomic annotation, multiomics profiling, single‐cell and spatial context, functional validation, and prospective clinical evidence. Continued efforts to integrate genomic discovery, mechanistic investigation, and clinical validation will be essential to fully realize the potential of variant interpretation in improving our understanding and management of HCC.

## Author Contributions

Y.Y., Z.X., and J.W. performed the literature review and drafted the manuscript. C.C. and Y.P. contributed to data interpretation and critical revision of the manuscript. B.L. conceived and supervised the study and provided overall guidance. Yuntao Ye, Zhulin Xu, and Jiang Wang contributed equally to this work and share first authorship.

## Funding

This work was supported by the Sichuan Provincial Natural Science Foundation (Youth Science Fund Type B, Grant No. 2026NSFSC1937) and the Basic Research Project of the Luzhou Municipal Science and Technology Bureau (Grant No. 2025LZXNYDJC13).

## Disclosure

All authors approved the final version of the manuscript.

## Conflicts of Interest

The authors declare no conflicts of interest.

## Data Availability

Data sharing is not applicable to this article as no datasets were generated or analyzed during the current study.
